# Sub-wavelength lateral detection of tissue-approximating masses using an ultrasonic metamaterial lens

**DOI:** 10.1038/s41467-020-19591-2

**Published:** 2020-11-24

**Authors:** Ezekiel L. Walker, Yuqi Jin, Delfino Reyes, Arup Neogi

**Affiliations:** 1 Echonovus Inc, Denton, TX 76205 USA; 2 Department of Physics, University of North Texas, Denton, TX 76201 USA; 3 Faculty of Science, Autonomous University of the State of Mexico, Campus “El Cerrillo”, Toluca, C.P. 50925 Mexico; 4 Advanced Materials and Manufacturing Processes Institute, University of North Texas, 3940 North Elm Street, Box Q, Discovery Park Annex, Denton, TX 76207 USA

**Keywords:** Ultrasound, Biomedical engineering, Acoustics

## Abstract

Practically applied techniques for ultrasonic biomedical imaging employ delay-and-sum (DAS) beamforming which can resolve two objects down to 2.1*λ* within the acoustic Fresnel zone. Here, we demonstrate a phononic metamaterial lens (ML) for detection of laterally subwavelength object features in tissue-like phantoms beyond the phononic crystal evanescent zone and Fresnel zone of the emitter. The ML produces metamaterial collimation that spreads 8x less than the emitting transducer. Utilizing collimation, 3.6x greater lateral resolution beyond the Fresnel zone limit was achieved. Both hard objects and tissue approximating masses were examined in gelatin tissue phantoms near the Fresnel zone limit. Lateral dimensions and separation were resolved down to 0.50*λ* for hard objects, with tissue approximating masses slightly higher at 0.73*λ*. The work represents the application of a metamaterial for spatial characterization, and subwavelength resolution in a biosystem beyond the Fresnel zone limit.

## Abstract (web summary)

Traditional methods for ultrasound detection in biomedical application suffer from limited lateral resolution. Here, the authors show that a phononic metamaterial lens can be used for spatial characterisation of subwavelength objects, even beyond the Fresnel zone of the emitting transducer.

## Introduction

Ultrasound serves as a popular imaging and therapeutic modality due to its relative non-toxicity, practical mobility, and low cost. In both non-destructive and medical applications, the ability to control the spatial sound pressure field in a material is critical for evaluation. These evaluations are used for diagnostics, with diagnostic accuracy from an image directly dependent on an accurate characterization of the size, shape, and contrast of materials comprising a composite^1^. Images that enjoy high fidelity of the distribution of materials with varying contrast are generally considered of higher quality. Ultrasonic imaging quality is both dependent on the minimum sizes detectable by a particular system, fundamentally a function of wavelength, and the minimum contrast detectable. Contrast and spatial reconstruction of a signal to produce an image is dependent on achieving high signal-to-noise ratios by controlling focusing, and reducing multiple scattering effects^2^.

The maximum imaging quality achievable with ultrasound is dependent of a variety of factors. Transducer frequency, frequency bandwidth, numerical aperture, depth to an object, and spatial sound pressure field, particularly focusing, dictate the maximum imaging quality capable by any setup^2,3^. In addition, ultrasonic systems do not maintain equal resolution along the axial and lateral directions. Whereas axial resolution is limited by the frequency and bandwidth both in theory and practice, the limitations of lateral resolution at any depth are defined by the lateral width of the beam^2^. All of these factors are encompassed by the term beamforming and work in tandem with signal processing to extract the maximum information from backscattered sound waves^4^. Though both axial and lateral resolution are critical for imaging, we focus specifically on lateral resolution.

A large proportion of current ultrasonic imaging systems utilize phased and linear array emitters combined with beamforming and signal processing techniques. Various metrics are used in literature to quantify the resolution of a system including full width at half maximum (FWHM) and minimum separation detectable (MSD) between adjacent objects^5–8^. For single element emitters, the FWHM for a focused Gaussian beam is defined as ${\mathrm{FWHM}} \approx \frac{{1.06\lambda F}}{a},$where *F* is the focal length, *λ* is the wavelength, and *a* is the aperture^9^. However, multi-element emitters comprise the bulk of ultrasonic biomedical imaging modalities used in practice and incorporates beamforming. Beamforming is particularly important as it encompasses how waveforms are generated to ultimately produce images.

Delay-and-sum (DAS) beamforming methods comprise the bulk of applied techniques with maximum MSD of ~2.1*λ* and FWHM commonly approaching $\sim\! 4.6\lambda \,$^2,5,10^. Other developing techniques include the synthetic aperture (SA)^6,11,12^, minimum variance (MV)^10^, Bessel beams^13^, and pixel-based beamformer (PBB) approaches^14,15^. All provide superior resolution to DAS under ideal conditions with resolutions reaching as high as *λ*/12 for FWHM using MV. However, in more practical settings, these techniques result in much smaller improvements over DAS with FWHM (MSD) of 0.38*λ* (0.91*λ*) for MV^10^, 3.3*λ*(2*λ*) for SA^11^, and 1.02*λ* (1.43*λ*) for PBB^15^. The maximum resolution of each technique occurs at the focal point, varies strongly with applied frequency, and must be within the acoustic near-field of the emitters. In addition, the application of the techniques in practice do not enhance imaging quality significantly^5,10,11^.

Phononic crystals are periodic structures comprised of two or more materials of differing physical properties where at least one of the materials, termed the scatterer, causes scattering from an incident sound wave or vibration. Based on the size, shape, arrangement of the scatterers, bandstructures arise that are analogous to those found in electronic and photonic crystals^16^. Within the first transmission band, the range of linear dispersion allows a phononic crystal to operate as an effectively homogenous medium. However, above this homogenization limit, the phase and group velocity of a transient wave may not coincide, leading to effects such as hyperbolic and elliptical metamaterials^17^, “slow” sound^18^, an effective index of refraction below 1.0, and even effectively negative index of refraction^19^. Dispersion above the homogenization limit may also be highly non-linear as a function of incidence angle allowing for anomalous gradient effects only demonstrated in these artificial materials^20^. These effects have been used to create filters^21^, waveguides^22^, effective media that operate as lenses^20^, and even thermal resistors^23^. Of particular interest are phononic metamaterials which maintain properties that aren’t observed in nature. These materials have formed tunable controllers for elastic waves^24^, mechanical cloaks^25^, Weyl materials^26^, switches^21^, tweezers^27^, and negative index lenses amongst others^17,18,28,29^.

Whereas phased-array approaches to high resolution depend almost exclusively on electronic signal processing, super resolution has been demonstrated with phononic metamaterials without the use of such. Holey structures, coupled bound modes, and negative index flat lenses have all seen use as conduits to super resolution as high as λ|50^30^. Metamaterials have also been used to create custom group velocity dispersion functions to temporally compress phonons for super resolution^31^. However, the super resolving power is generally restricted to the evanescent near-field of the device or within a few wavelengths of the surface, making the structures impractical for many applications^32^.

The use of metamaterial lenses (ML) for material characterization, specifically in biomaterials, has never been demonstrated^30^. Where in this text, *tissue phantom* references gelatin tissue phantom^33^ that approximates a tissue-like biomaterial, we demonstrate the use of a ML to examine a material beyond the evanescent near-field, demonstration of subwavelength detection of masses in a tissue phantom, and enhanced lateral resolution in tissue phantoms beyond the Fresnel zone of the emitting transducer. The ML produces an approximately collimated ultrasonic beam that is both laterally small and maintains low beam spreading along the optical axis. The collimation-like effects extend past the theoretical near-field of the transducer, and demonstrates strong side-lobe suppression similar to Bessel-beams^13^. Lastly, the lens is used with gelatin tissue phantoms containing embedded objects of varying sizes to approximate practical application in non-ideal systems. The results demonstrate the feasibility of the lens for improving lateral imaging quality based on increased resolution.

## Results

**Fig. 1:**
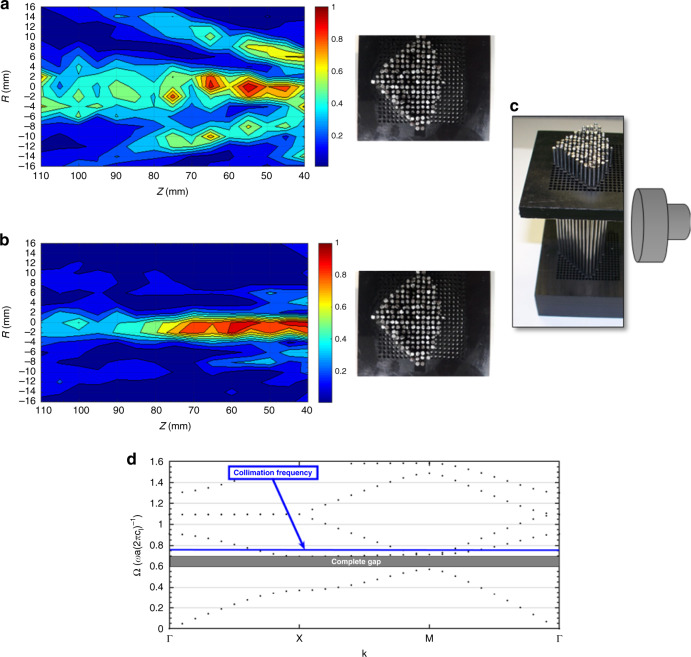
Metamaterial lens with sound field at collimation and non-collimation frequencies. Experimentally measured spatial sound field of metamaterial lens at 527 kHz demonstrating beam divergence (**a**) and 576 kHz demonstrating collimation (**b**). **c** Image of 2D phononic crystal metamaterial lens used for examining enhanced lateral resolution with the setup for the emitter. **d** The collimation frequency occurs above the first complete gap where $\frac{{\mathrm{d}k}}{{\mathrm{d}{\mathrm{{\Omega}}}}}$ is highly variable and non-positive for some wavevectors, indicative of metamaterial features.

The ML is a 2D square lattice phononic crystal of stainless-steel rods in water ambient (Fig. 1). For this lens, collimation only occurs for narrow, limited bands of frequency in the second band of the crystal, far above the homogenization limit. In addition, the lens does not function as described in the axis parallel to that of the cylindrical scatterers. Outside these bands of frequencies, the ML causes the transient beam to diverge. As seen in Fig. 1a, the beam diverges strongly at 527 kHz, but remains relatively collimated for the length of the examined area (27λ) for 576 kHz (Fig. 1b). Figure 1c depicts the 2D ML and relative location of the emitting transducer. The dispersion curve of the lens is given for reference (Fig. 1d). The collimation occurs above the first gap and homogenization region which signifies metamaterial behavior.

The emission source was a single element, unfocused, planar ultrasonic transducer. For ultrasound, the length of the Fresnel zone is also termed the near-field. Near-field length, *L*, is defined as $L = \frac{{a^2}}{{4\lambda }},$ where *a* is the aperture of the transducer, and λ is the wavelength of interest. At the collimation frequency for the ML at 576 kHz, *L* = 58.4 mm. With the ML, collimation occurs from 42 to 112 mm with the beam spreading 4.5% in the area examined. Axially, the highest measured signal intensity with the narrowest beam-waist was detected at ~67 mm, 3.3λ past the Fresnel zone. The occurrence of a narrow beam-waist beyond the Fresnel zone is significant as a focused transducer can only achieve its minimum beam-waist width well within the Fresnel zone limit. Thus, the ML does not sacrifice depth while maintaining a narrow beam-waist.

For the frequency regions of collimation, the measured FWHM reduces from 11 mm (4.27λ) in water without the lens to ~3.3 mm (1.28λ) with the lens. The Rayleigh length provides a good measure of expected beam divergence, and is defined as $z_r = \frac{{\pi w_0^2}}{\lambda }$, where *w*_*o*_ is the radial beam waist. Based on measured results, the Rayleigh length for the transducer alone (TA) is *z*_TA_ = 14.3*λ*, and *z*_ML_ = 1.29*λ* for the ML. The ML demonstrates strong collimation as the beam diverges only 4.5% over the 27*λ* measured which is an order of magnitude larger than its calculated Rayleigh length. In addition, as seen in Fig. 1b, the ML does not maintain significant side lobes. The combination of collimation, narrow beam-waist, extension past the near-field, and low side lobes made the ML particularly intriguing for improving lateral resolution.

### Water ambient

**Fig. 2:**
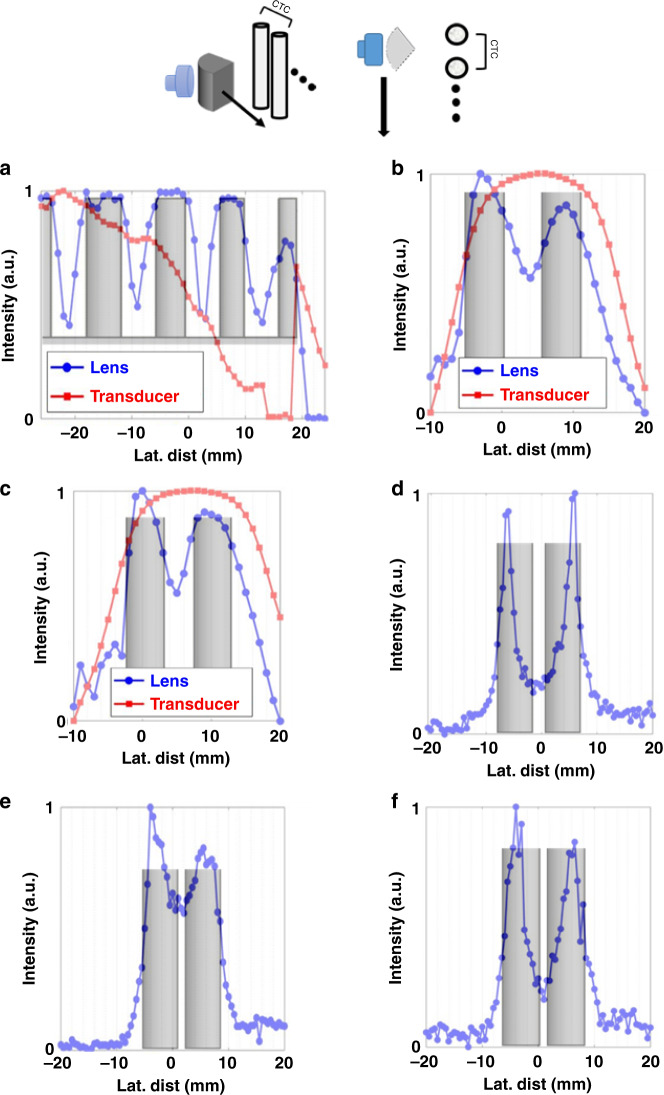
Monostatic characterization of lateral resolving powers of metamaterial lens in water ambient with ABS 3D printed plastic rods. Intensity is stated for all in arbitrary units (a.u.). **a** Sample of equally spaced rods of decreasing diameter. Transducer alone does not clearly detect any of the rods. **b**, **c** Scan of 5 mm diameter rods with gaps of 5 mm (**b**) and 4 mm (**c**) between the boundaries. Only the ML shows the presence of more than one object. **d**–**f** Rods with subwavelength gap spacing of 0.69*λ* (**d**), 0.47*λ* (**e**), and 0.41*λ* (**f**).

The performance of the ML was evaluated in both water ambient and tissue-approximating phantoms. Lateral scans of the samples were as illustrated in Fig. 2. In water, the FWHM of the collimated beam maintained a lateral FWHM of ~1.28*λ* (Fig. 1b). To compare the relative performance of the ML to the transducer in water, two samples of 3D printed rods were laterally scanned and the results plotted in Fig. 2. Sample 1 (S1, Fig. 2a) was a single 3D printed sample of four cylindrical rods of decreasing size spaced at equal intervals. Sample 2 (S2, Fig. 2b–d) consisted of five pairs of rods of equal size, spaced at decreasing distances. Detailed descriptions of S1 and S2 is given in the “Methods” section. Though both samples provide tests for resolution, S1 is ideally suited to compare acuity, and S2 to lateral resolution as it pertains to minimum separable distance. For this work, we define acuity as the ability to detect a single object and lateral resolution as the ability to detect the correct number of objects in close proximity.

Acuity and lateral resolution for the emitting TA was too low to distinguish any of the rods individually in S1 (Fig. 2a). The transducer shows an overall signal decrease due to the decreasing overlap of the cross-sectional area of printed ABS rods with the ultrasound beam. With the ML, each rod is clearly able to be distinguished as the signal shows a sharp decrease at the edges of each rod (Fig. 2a). Using the value of half a peak’s intensity to determine a boundary, the ML finds rod widths of 8, 7.5, 6, and 4 mm for the rods compared to caliper measured values of 6, 5, 4, and 3 mm for S1. Based on the distance between the minima, the rods have a center-to-center (CTC) distance of 12, 10, and 10 mm which compares favorably with measured values of 12, 11, and 10 mm.

The ML capability to resolve two distinct objects in close proximity was evaluated with S2. The 5-mm wide rod pairs had spacing between neighboring boundaries ranging from 1.93*λ* down to 0.44*λ*. The TA was not able to resolve two distinct objects in any case, even for the case where the rods were spaced larger than 1*λ* (Fig. 2b, c). For all pairs, the ML was able to resolve two separate objects. Using the same standards as S1 to evaluate size and CTC separation, we found the rods spaced 5 mm (CTC 10 mm) to be 6 and 7 mm wide with a CTC of 12 mm using the ML. For the rods 1 mm closer (CTC 9 mm), rod widths were measured as 5 and 7 mm with a CTC of 9 mm. However, the ML was not able to resolve either the correct size or CTC distance between the objects that were spaced sub-wavelength.

The limit of the resolving power of the ML in water ambient was tested with S2 by scanning rods with neighboring boundaries closer than 1*λ*. Three samples were examined with 1.9 mm (0.74*λ*), 1.3 mm (0.50*λ*), and 1.14 mm (0.44*λ*) gaps between the rods or CTC values of 6.9, 6.3, and 6.14 mm (Fig. 2d–f). In each case, the ML was not able to accurately resolve the rod widths and separation. For the 6.9 mm CTC rods, the rods measured 2.5 and 2 mm in width with a CTC of 12 mm. The rods with a CTC of 6.3 mm were found to be 3.5 mm wide each with a CTC of 10.5 mm, and the last pair of rods examined (6.14 CTC) had widths of 3.5 mm and a CTC of 10.5. Despite the inaccuracies, the ML was clearly still able to resolve two distinct objects in all cases with a sub-wavelength lateral gap.

### Gelatin tissue-approximating phantoms

Gelatin tissue phantoms have served as sufficient human tissue substitutes for the evaluation of ultrasound and other effects^34^. Both bistatic and monostatic arrangements were used to evaluate the performance of the ML in gelatin tissue phantoms. The tissue-like phantoms were made following the procedures of ref. ^33^. Both hard objects and tissue approximating objects of varying size and composition were synthesized into the phantoms and the characterization capability of the ML and TA compared.

**Fig. 3:**
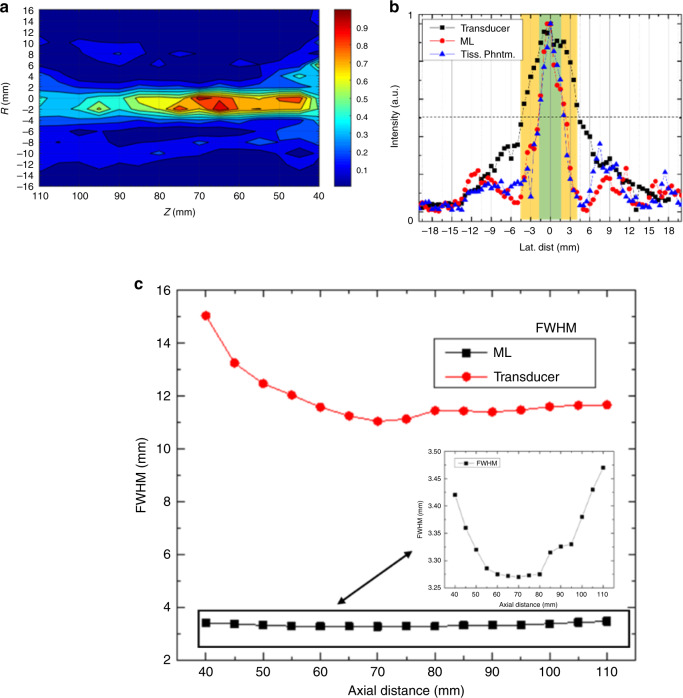
Illustration of collimation behavior of the ML. Collimation-like spatial sound field of ML at 576 kHz (**a**) and lateral beam width of the emitting transducer compared to the lens in a gelatin tissue phantom (**b**). The tissue phantom does not cause beam divergence and the beam remains collected along the optical axis. Whereas FWHM of the beam varies by 36% for the transducer, it only varies 4.5% for the length of the beam for the ML (**c**).

We investigated the effect of gelatin tissue phantoms on beam divergence by placing a 60-mm thick gelatin phantom sample in front of the ML and TA and measuring the spatial beam width at the back of the sample using a needle hydrophone. The measured beam profile from the TA for collimation is shown in Fig. 3a. The FWHM of the TA is 11 mm while the width of the ML beam is nearly equal for both the tissue-like phantom and water at ~3.3 mm (Fig. 3b), comparing favorably with results in water only (Fig. 1b). The FWHM for the ML diverges 4.5% in the area examined versus 36.5% for the standalone transducer (Fig. 3c).

**Fig. 4:**
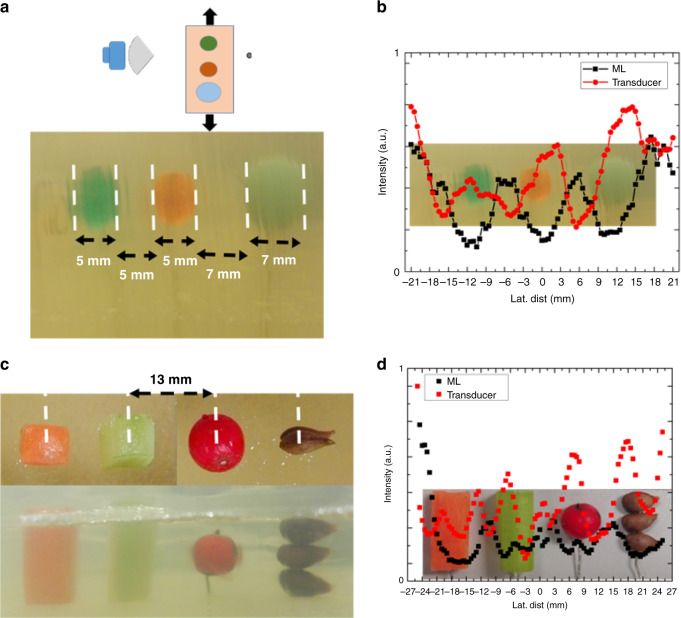
Bistatic detection of objects in gelatin phantom using ML. **a** Spherical plastic beads of 5, 5, and 7 mm in a gelatin tissue phantom spaced 5, 5, and 7 mm apart at a depth of 30 mm. **b** Recorded scan of the beads with the transducer and lens in a bistatic arrangement overlayed with the known position of the beads in the tissue-approximating phantom. **c** Biomasses of celery, carrot, Yaupon berry, and apple seed to simulated masses in tissue. **d** Scan of biomasses overlayed with known position of masses at frequency of collimation.

Both Sample 3 (S3, Fig. 4a) and Sample 4 (S4, Fig. 4c) were examined using a bistatic setup where the gelatin tissue phantom with the embedded objects was swept between the ML and a needle hydrophone detector. Sample 3 (S3) consisted of three hard, spherical beads 5, 5, and 7 mm in diameter with lateral spacing of 5 mm between the 5 mm beads and 7 mm between the 5 and 7 mm beads. Figure 4a shows both the S3 sample, and illustrates the bistatic scan. Additional details on S3 synthesis are given in the “Methods” section. As in the case for S1 and S2, comparison is given between the TA and ML.

Based on the number and location of minima, the TA indicates the presence of four hard objects in S3 (Fig. 4b). For convenience, an image of S3 was overlayed onto the measured spectra based on the known position of S3 during the experiment. The two neighboring 5 mm objects are not clearly distinguishable from each other, indicative of the spacing and size of the objects being near or below the resolution limit of the TA. Another object, ideally the 7 mm bead, is clearly represented in the transmission data. However, combination of the known locations of the beads with the recorded data show the TA to be inaccurate in both the size, position, and number of the inorganic objects. As is expected, the relatively large beam waist of the TA causes it to have a low resolution of the beads.

The use of the ML to detect and characterize the presence of the beads provided greater clarity than the TA in S3 (Fig. 4b). Combination of the measured transmission versus position with the known position of the objects yields a representative picture of the lateral position and sizes of each representative bead. The sizes of the beads using the ML are 6 mm, 5.5 mm, and 7.5 mm with a CTC of 11 mm for each of the neighboring beads. The measurements compare favorably to the known diameters of the beads, while the CTC results are +1 mm for the neighboring 5 mm beads and −2 mm for the neighboring 5 and 7 mm beads. The front and back surfaces of the tissue phantom were not perfectly planar, contributing to the measurement errors.

We examined the ability of the lens to detect organic materials by placing four vegetable tissue mass objects of differing compositions and shapes in the tissue-approximating phantom. Fruit and vegetable tissues have been used to approximate masses and cysts in other works^34^. Sample 4 (Fig. 4c, S4) is a gelatin phantom containing a 7 mm × 6 mm × 16 mm rectangular prism of baby carrot (*Daucus carota)*, a 6.5 mm × 6 mm × 16 mm rectangular prism of celery (*Apium graveolens*), three stacked, tear-drop shaped, red apple seeds (*Pyrus malus L*.), and an 8 mm diameter Yaupon berry (*Ilex vomitoria*). The selection of the samples was based on the variety of material compositions, object shapes, and their use as approximation of tumorous masses in tissue^33^. In similar fashion to the hard beads, the masses were placed into the gelatin tissue during the synthesis process with a CTC distance of 13 mm between neighboring objects.

As in the case of the beads, the TA does show the presence of objects, but the size, number, and position of the objects does not correlate with the known size, number, and position of objects (Fig. 4d). Our measurements using the ML, however, detected the size and positions of each of the masses relatively accurately as shown in Fig. 4d. Though the CTC distance between neighboring objects was set at 13 mm, the gap between object boundaries ranges is ~6 mm (2.17*λ*). The estimated cross-sectional sizes from the ML are 9 mm for the rectangular masses, and 8.25 mm width for the Yaupon berry. Visual inspection of the figure shows good agreement between the width of the apple seeds and the transmission spectra. In addition, both the celery and Yaupon berry show slightly decreased transmission at their centers. The Yaupon berry is hollow and filled with air, while the celery cut was centered around the core of a stalk which contains pithy tissue. Figure 4d indicates the presence of both at the centers of each mass.

**Fig. 5:**
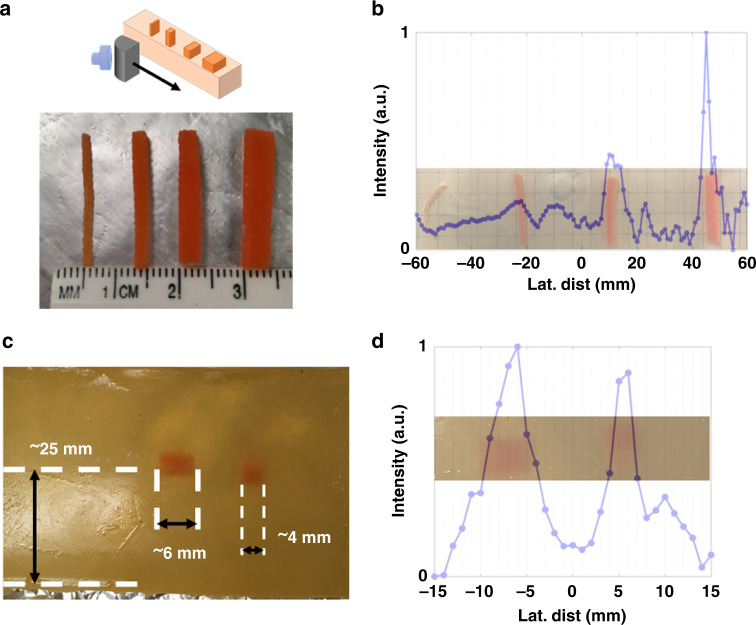
Monostatic scan of organic masses in gelatin tissue phantom for acuity and lateral resolution. **a** Carrot tissue masses with lateral widths of 1–4 mm in 1 mm intervals. **b** ML lateral scan of biomasses overlayed with image of the sample. Samples larger than 0.72*λ* are detected. **c** 6 mm and 4 mm rectangular masses in tissue phantom with lateral size and spacing > 1*λ*. **d** Lateral scan of clearly shows the objects when overlayed with an image of the sample as known in the measurements.

Practically applied ultrasound utilizes a monostatic arrangement where the emitter simultaneously serves as the detector. In addition to the bistatic analysis, we also examined the acuity and lateral resolution of the ML using a monostatic setup. All monostatic samples consisted of carrot tissue “masses” embedded in gelatin tissue phantoms. Similar to the case for biostatic measurements, the lens remained stationary while the samples were swept laterally using a translation stage. An illustration of the sweep is indicated in Fig. 5a. Samples 5 and 6 were primarily utilized to examine ML acuity, while Sample 7 was used to investigate lateral resolution as it pertains to minimum separable distance detectable.

Though synthesis details are presented in the “Methods” section, a brief description of each sample is given in this section to aid the reader in interpreting results. Sample 5 (S5, Fig. 5a, b) consisted of four carrot mass slivers cut to widths of 1, 2, 3, and 4 mm spaced >30 mm, and Sample 6 (S6, Fig. 5c, d) of two carrot masses 6 mm × 4 mm × 4 mm and 4 mm × 4 mm × 4 mm separated by 8 mm CTC. For the 1 mm sliver, the heat of the gelatin solution caused the shape to distort during synthesis.

Our findings for the ML in S5 are of mixed results. For Sample 5, the ML spectra detects all masses ≥2 mm in width with the 2-mm mass showing the weakest contrast when compared to the larger biomasses. In addition to the biomasses, an artifact from the synthesis container is also correctly found between the 2 and 3 mm carrot slivers. Though the positions of the masses are accurately represented, the sizes of the biomasses as determined using the FWHM of a peak are inaccurate. For S5, lateral sizes of 7, 7, and 5 mm were found for the 2, 3, and 4 mm slivers (Fig. 5b). For S6, both masses are clearly indicated by the ML, with a derived width of 6.5 mm for the 6 mm object and 3.5 mm for the 4 mm object (Fig. 5d). In both S5 and S6, the CTC between masses found with the ML agree well with known CTC spacing. While Fig. 5b demonstrates the capability of the ML to detect the presence of biomass down to 0.73*λ*, accurate resolution of the lateral biomass size only occurs when it is >1.46*λ* (Fig. 5d).

**Fig. 6:**
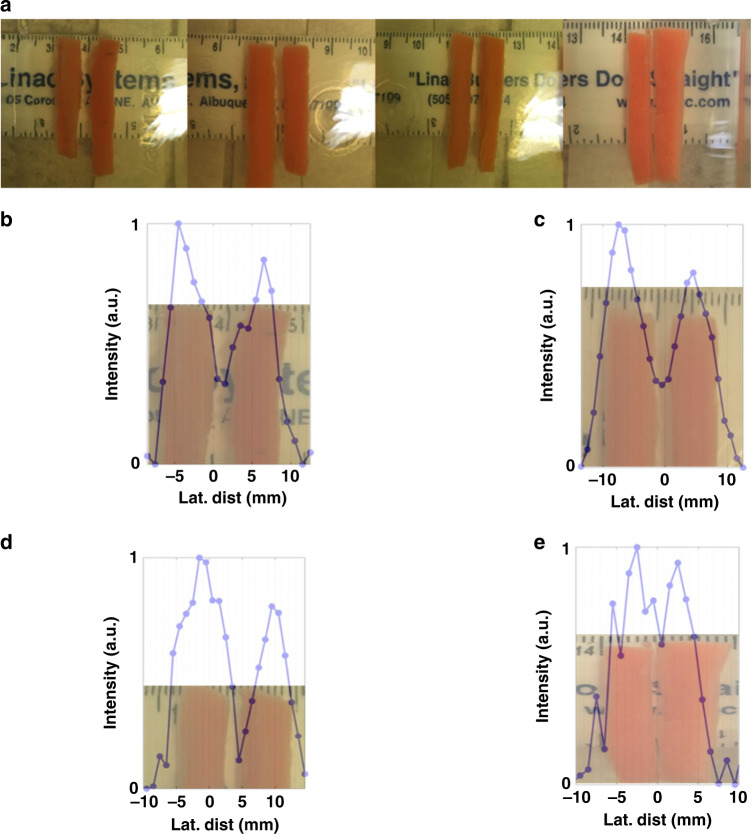
Examination of minimum separable distance of tissue masses. **a** Four pairs of 7 mm wide carrot tissue masses spaced 4, 3, 2 mm, and 1 mm apart. The scanned results are overlayed for each gap of 1.45λ (**b**), 1.09λ (**c**), 0.72λ (**d**), and 0.36λ (**e**).

Our final sample was used to investigate the minimum separable distance detectable for paired biomasses using the ML. Four pairs of carrots ~7 mm in lateral width were embedded in the same tissue to make Sample 7 (S7, Fig. 6a). The pairs were spaced such that the neighboring boundaries had an average separation of 4 mm (1.44*λ*), 3 mm (1.08*λ*), 2 mm (0.73*λ*), and 1 mm (0.36*λ*). The lens was swept over the sample and the reflected signal recorded identical to the techniques used for S5 and S6.

The results show the ML able to clearly discern the presence of two masses down to a 2 mm gap (Fig. 6b–d) with relatively weak indication of two objects at 1 mm spacing (Fig. 6e). For the pairs ≥0.73*λ* apart, the average width of the individual carrot biomasses was found to be 7 ± 1 mm. At 1 mm, overlaying the recorded data with the image and known position of the biomasses shows the ML distinguishing two objects. However, the low contrast in the signal alone would only lead to positive detection of the two biomasses if the criteria were relaxed for evaluating objects widths using FWHM.

### Direct comparison with focused transducer

Ultrasound detection or imaging commonly uses emission sources that can focus. Focusing is achieved using phase-delay pulsing in phased/linear array transducers, curved emission surfaces, or lenses. All eventually result in narrowing of the beam-waist, thus improving lateral resolution. However, as focusing serves to reduce the distance between the emission sources and the Fresnel transition zone, resolving power at depth is sacrificed for absolute resolution, where peak resolving power occurs in the focal zone^35^. Minimum beam width is achieved when the focal length is a minimum for an aperture size.

**Fig. 7:**
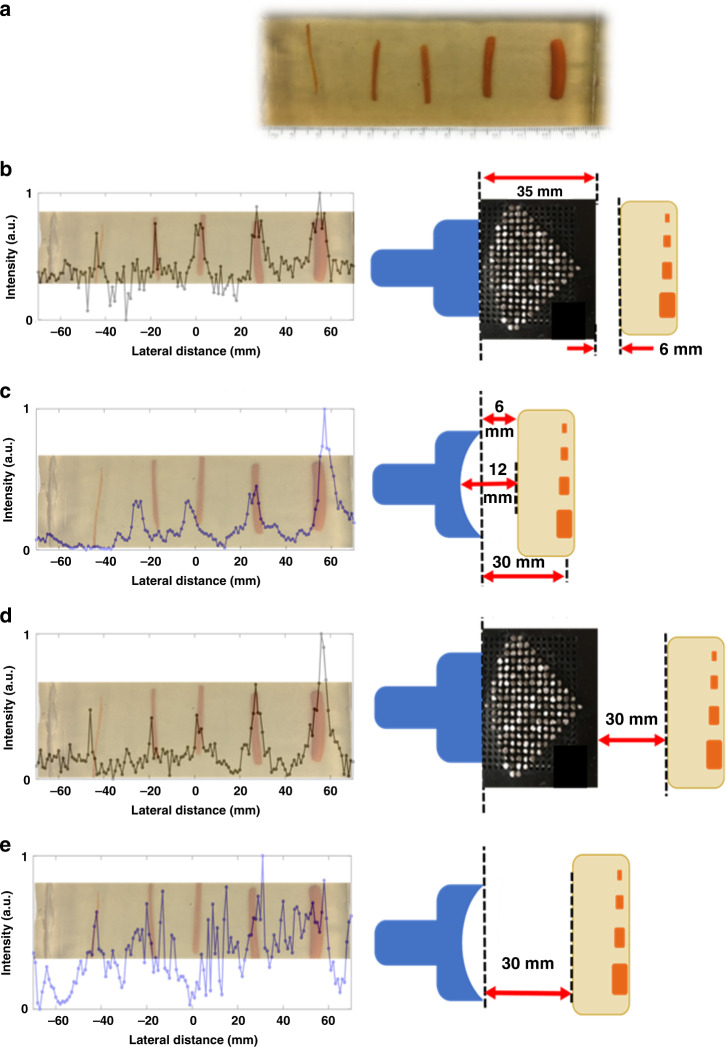
Comparison between a focused transducer and the ML both at and beyond the focal zone. **a** Samples of carrot tissue ranging from 1.5 mm (left) to 6 mm (right) in width. Scan with carrot tissue at the focal length of the focused transducer for the ML (**b**) and focused transducer (**c**). Scan with carrot tissue in the Fresnel transition zone of the unfocused transducer for the ML (**d**) and focused transducer (**e**). Comparative acuity of ML vs. focused transducer at (**b**, **c**) and beyond (**d**, **e**) the transducer focal length. Scans were performed monostatically with carrots at average depth of 15 mm in the tissue.

To this point, evaluation and comparison have centered on comparison between the ML and the planar TA at the Fresnel zone limit, demonstrating resolution at depth. Sample S8 was synthesized in likeness to Sample S5, where five carrots tissues are placed 15 mm into a 22-mm thick gelatin tissue phantom spaced 27 mm apart on average (Fig. 7a). Carrot widths are 1.5 mm (0.58*λ*), 2 mm (0.73*λ*), 3 mm (1.07*λ*), 4 mm (1.44*λ*), and 6 mm (2.33*λ*). It should serve as note that the smallest carrot shifted position in synthesis such that it was only 5 mm into the tissue phantom, instead of 15 mm.

An Olympus 1”, 0.5 MHz V301-SU focused transducer with a focal length of 31.75 mm was then used to evaluate acuity in comparison with the ML. FWHM of the focused beam was calculated as 3.31 mm (1.28*λ*) in the tissue-like phantom from the equipment manufacture standard calibration technical notes on the transducer. The FWHM is the minimum achievable with the single-element focused transducer and maintains the shortest focal length. Unlike the ML, the focusing with the transducer is conical versus two-dimensional. Measurements were taken such that the scattering tissues were in the focal zone of the transducer, where maximum lateral resolution is achieved, and outside the focal zone, within the unfocused near-field limit.

At the focal length, the carrot masses are 30 mm (11.6*λ*) from the focused transducer and the ML. The calculated minimum beam waist for the focused transducer only occurs for a concentrated region, but is comparable to the FWHM of the ML. Figure 7 shows the results. In the focal zone (Fig. 7b, c), the transducer detects all but the smallest carrot mass. FWHM finds each carrot to be 4.5, 6, 6, and 8 mm, with CTC distances of 27 mm on average. Here it is interesting to note that the 1.5 mm wide carrot is not detected or resolved as the shift in its position by 10 mm places it outside of the focal zone (Fig. 7b). However, when the sample is displaced by 30 mm, the acuity of the focused transducer is sufficient to detect the 1.5 mm carrot, while being insufficient to detect any other masses (Fig. 7d, e). When the smallest mass is detected, its FWHM is found to be 2.5 mm by the focused transducer. Conversely, in all cases the ML is able to resolve all masses, with FWHM for the masses of 1.5, 1.75, 3.75, 5.25, and 7 mm and CTC average of 24 mm. All dimensions are improvements over the focused transducer to varying degrees with some limitations due to data being acquired every 0.5 mm.

## Discussion

Spatial collimation is a feature of the Fresnel zone of the transducer. The ML significantly reduces the beam-width while maintaining a narrow, weakly diverging beam significantly longer than the Rayleigh length. This effect was termed funneled focusing in^36^, where the ML was first proposed in a numerical model. The experimentally measured low divergence of the ML beam as compared with the Rayleigh length combined with the extension of the beam beyond the acoustic near-field length of the emitter lead to our conclusion of revising the term to funneled collimation.

In agreement with^36^, the funneled collimation achieved by the ML only occurs for a small set of frequencies in the second transmission band of the base phononic crystal. The low lateral beam width and spatial dispersion serve as ideal properties to demonstrate enhanced lateral resolution beyond the acoustic near-field of the emitter. In prior works, the design of the lens was discussed, but never demonstrated in practice^36^. Here, the low lateral beam width and lack of spatial dispersion was confirmed with measurements of the sound field. At frequencies where collimation occurred, FWHM beam widths of 1.28*λ* were achieved and persisted with minimal beam spreading (~4.5%) for the extent of the examined area (28*λ*). Unlike beamforming techniques, the ML did not exhibit strong side-lobes at the frequencies of funneled collimation. As compared with the TA, both in water and in tissue phantoms, the ML demonstrated significantly improved acuity and lateral resolution without additional signal processing techniques.

The acuity of an imaging systems determines the extent to which an object’s size can be determined. In the present work, we examined both acuity and lateral resolution without the aid of additional signal processing, instead focusing on the raw FWHM transmission characteristics to derive information. Water served as a baseline from which the ideal limitations of the ML and TA could be associatively analyzed and compared to other techniques. FWHM and MSD are metrics used for standard signal processing in ultrasonic imaging. DAS beamforming is the primary method used for ultrasonic imaging systems, with a minimum MSD of 2.1*λ* and a FWHM of roughly 4.6*λ*. Though acuity isn’t explicitly mentioned, here its importance is discussed due to the need for ultrasonic imaging modalities to distinguish material defects or tissue masses from surrounding material.

For water, the reduction of the FWHM from the measured 4.27*λ* for the emitter at the near-field limit to 1.28*λ* at and beyond the same point strongly impacts the acuity and MSD in water. For the plastic rods examined in S1 and S2, the TA was not able to detect any independent objects for either due to the large FWHM of the TA beam. In S1, the scattering cross-section of the rods decreases with rod diameter resulting in the continuously decreasing signal associated with the TA (Fig. 2a). For S2, the overall scattering cross-section slightly increases as the rods decrease in proximity while maintaining a constant diameter (Fig. 2b). Regardless, the TA cannot resolve the rods due to the large beam-waist. The maximum CTC distance between objects was 6 mm (2.17*λ*) with boundaries between the rods lower. Though DAS beamforming has not demonstrated the ability to resolve similarly sized objects in other works, other advanced beamforming techniques such as MV and PBB have FWHM and MSD that indicate they would suffice under ideal conditions^5,11^.

The lower FWHM of the ML in water was measured to extend from the end of the lens past the acoustic near-field limit. This collimation enhances resolution over the TA without additional signal processing in water. For S1, each of the rods were clearly able to be resolved and correctly located in its position in water. Though the acuity shows maximum errors exceeding 30% of the known size (4 mm measured vs. 3 mm actual), the ML still maintained the acuity to detect the smallest rod of S1 which was 3 mm (1.09*λ*). As the scattering, cylindrical rods both maintain spacing and dimension comparable with the incident wavelength, the FWHM used to determine size is subject to Rayleigh diffraction introducing errors. However, whereas the dimensions of each object are dependent on the recorded intensity profile, CTC distances are taken from central maximum. Here, the CTC for sample one was of greater accuracy with maximum error of ±1 mm.

For the lateral spatial resolution, correlated with the MSD, the decreased lateral beamwidth and apparent collimation of the ML also greatly improves detection of the pairs of rods in S2. For S2, the 5 mm diameter rods were spaced more than 1*λ*, the lateral diameters were found within 2 mm of their known size and CTC distance. For the S2 pairs spaced below 1*λ*, the ML was able to clearly detect the presence of two distinct objects, but did not accurately find the size or separation using the same standards as other samples. Despite this shortcoming, the ML was able to resolve two objects with a gap of 0.41*λ* without any additional signal processing in a monostatic arrangement. These results compare favorably with all the beamforming techniques except MV, though MV lacks resiliency to less than ideal applications. Unlike other techniques, however, sub-wavelength detection occurred beyond the acoustic near-field. The authors are unaware of any other techniques that have achieved such detection capabilities beyond the Fresnel-zone limit, particularly without advanced post-processing.

Both S2 (Fig. 2b–f) and S7 (Fig. 6) were used to examine the lateral resolving capabilities of the ML. However, the thrust of S2 was to establish a likely upper bound of resolution of the ML by using ideal conditions. Both contain pairs of objects at the edge of the Fresnel-zone with decreasing gap spacing between the paired objects. For S2, the objects are inorganic, hard, well-defined in shape, and in DI water, an ideal medium. For S7, the vegetable tissue masses are cut rectangularly, but maintain some irregularity in shape due primarily to heat deformation from the gelatin synthesis process. The presence of vegetative masses in the gelatin phantom approximates organic foreign bodies in soft tissue. Ultrasound is particularly suited for detection of organic foreign bodies in practice as detection with other modalities remains challenging^37,38^. Frequencies near 10 MHz, roughly 20× that of the present work, have been utilized to sonographically detect organic foreign bodies down to 2.5 mm^37^. The evaluation of S7 with ML provides direct reference to capabilities of the ML in a setting which ultrasound is of increased importance.

In both water and gelatin tissue, sub-wavelength detection of the gap is demonstrated with varying degrees of accuracy on the object sizes and gap widths. Though both water and the tissue-approximating phantom have nearly identical measured speed of sound, they do have contrast in elastic properties. S2 shows slightly higher resolution than S7 primarily due to the presence of hard objects in water ambient, which have a larger impedance mismatch with water than vegetable tissue masses and gelatin tissue phantom. In S7, the lower elastic contrast between the carrot tissue and gelatin along with the slight irregularities in shape impact the back scattering from the edges of the sample objects, essentially blurring boundary edges of the masses. Similar behavior is observed in S3 and S4, where plastic beads are more accurately characterized than the various organic masses of S4.

Though metamaterials have been proposed for ultrasonic bio-imaging, it has not been demonstrated in practice. Prior works concerning imaging with phononic metamaterials, specifically those with subwavelength resolution, require coupling to the evanescent near-field of the phononic crystal^30,32,39^. Coupled bound modes allow for the reconstruction of subwavelength point sources^32,40–42^, or the detection of scattered evanescent waves^30^. Though these metamaterials demonstrate resolution beyond the Rayleigh diffraction limit, and indeed, beyond that of the ML, the necessity of coupling to the evanescent near-field means objects must be within a few *λ* of the phononic metamaterials to be imaged. The demonstrations of subwavelength imaging have, thus far, only been achieved using a bistatic arrangement with a separate emission source and detector^30^, or only reconstruction of point sources. The limitations of such systems make application of each in practice challenging. The ML is distinct in that the lateral resolution is achieved beyond the evanescent near-field of the lens, extends pass the Fresnel zone of the transducer, and is applied in a practical, non-ideal setting.

Here, the ML was applied to a gelatin tissue phantom commonly used for approximating healthy human tissue for both ultrasonic characterization and ballistics amongst others. In the samples, both inorganic and organic objects of various sizes were placed into the phantoms to approximate masses. The samples sizes and depths into the tissue phantoms were synthesized such that they accommodated available evaluation equipment in water ambient. In addition, unlike commercial ultrasound imaging systems that combine advanced signal processing with handheld application, the ML and sample were setup such that signal vs. position data were recorded with position controlled by a motorized translation stage. The tissue phantoms with various inclusions were made not with the motivation to be ideal, but to be equivalent comparisons between the ML and TA.

Bistatic measurements are not regularly used in practical application of ultrasonic imaging, but provide valuable additional information about the capabilities of the presented ML. Transit based imaging techniques are based on thru-transmission and lack the back-scattered reflections that cause additional errors in monostatic imaging arrangements. The bistatic arrangement was used to evaluate both hard objects and biomasses in tissue phantoms in samples S3 and S4. Both S3 and S4 were 30 mm into a 60 mm tissue phantom, but overall were near the Fresnel-zone limit of ~60 mm from the emitter.

The TA detected the presence of objects both in S3 and S4. However, the number, size, and positions of the objects, both for the biomass and hard objects was inaccurate. For the beads, the two 5 mm beads exhibited a strong overlap, making determination of the presence of two separate objects uncertain. The last 7 mm bead was clearly able to be detected, but the presence of all the objects did not align with the known positions of the objects during the measurements. For S4, the spectrum for TA strongly indicates the presence of more than 5 biomasses with the lateral positions of the objects shifted from their known position. All objects in S3 and S4 were >1*λ* and CTC and gap spacing exceeding 1*λ*.

For the ML, both the size, positions, and relative CTC are much more accurately recovered in the results. For S4, the sizes and positions of the beads were recovered within 1 mm of their known sizes and spacing. Though none of the objects were sub-wavelength in either spacing or size, the 5 mm objects were below the MSD limit of standard DAS beamforming. In other works, SA, MV, and PBB showed little practical improvement in resolution over DAS beamforming in tissue samples. DAS beamforming is ideally restricted to the near-field, with FWHM and MSD only reaching maximum effectiveness where focusing is achieved. For S4, the objects were at the near-field limit of the unfocused transducer, showing that the ML provides superior resolution at depth as compared to existing beamforming techniques.

Of particular interest is the application of ML to detect or image tissue masses in otherwise healthy tissue. This was explored in a bistatic arrangement in S4. The authors are not aware of any known works where metamaterials were applied to image or detect masses in biocomposites. Whereas the number, size, and position of the masses at the near-field limit were not accurately recovered by the TA, the ML shows a strong correlation to the position and size of the masses. For all biomasses, the gap and size of the fruit and vegetable tissues exceeded 2*λ*. The presence of each is clearly represented in Fig. 4. Our results found the sizes of regularly shaped masses of celery and carrots to be relatively accurate, within 1.5 mm of their known widths without any additional signal processing.

Ultrasound is primarily utilized with an emitter that also serves as a detector, a monostatic arrangement. Examination of samples with thru-transmission techniques often presents difficulties due to the size, shape, arrangement or location of the samples to be examined. Pulse-echo ultrasound spectroscopy forms the underpinnings of nearly all practically applied ultrasonic imaging modalities. The ability to accurately detect the size and positions of masses was further explored for the ML in the monostatic setup for samples S5 and S6.

In these samples, carrots were used as masses, where both samples concern the limitations of acuity. In S5, mass widths ranged from 1 to 4 mm in 1 mm intervals. The ML was not able to detect the 1 mm mass. However, it was able to clearly detect the 2–4 mm masses at their correct locations. For S5, subwavelength acuity is demonstrated in a practical settings as the width of the organic object is 0.72*λ*. In S6, both masses are longer than 1*λ* with widths that are within 0.5 mm of the known values. The smallest object in S6 was 4 mm (1.45*λ*) and was still accurately resolved in tissue phantom.

The S7 sample focused on the limitations on lateral resolution or MSD for organic masses in tissue-approximating phantoms. For gaps >1*λ*, the ML correctly detects the size of the objects and CTC distance between them. However, similar to the case for closely spaced objects in water ambient, though the ML is able to detect the presence of two distance objects with gaps subwavelength, the size and CTC distance of the objects are not accurately recovered using the uniform FWHM standards of the transmission dips. Still, without advanced signal processing techniques, the collimation at nearly 580 kHz can distinguish two separate objects down to 0.36*λ*, far below any known DAS beamforming works. In addition, the ML has comparable resolution to the most advanced signal processing techniques in samples with masses and objects that are less than ideal.

This work was performed at ~580 kHz, far below the 2+ MHz used in standard ultrasound imaging in practice. However, the use of phononic crystals allows for scaling to higher frequencies if the geometry of the setup is maintained. Where ${\mathrm{{\Omega}}} = \frac{{\omega a}}{{2\pi c_l}}$ is the reduced frequency that is referenced for periodic phononic structures^16^, and the collimation frequency is Ω ≈ 0.75, collimation occurs only at this reduced frequency for the present geometry and is proportional to the acoustic near-field. The dependence of the collimation frequency on the geometry results in an emission source aperture that is also dependent on the collimation frequency. Where the location of the minimum beam waist, $\zeta$, is considered the effective focal length of the ML, $\zeta [mm] \approx \frac{{40.2}}{{f\left[ {MHz} \right]}}$. An effective f-stop of ∼1.25 results from the ratio of $\zeta$ to the aperture. However, the collimation results in a depth of field of nearly 27*λ*.

Though axial resolution is not a focus of this work, it is a subject of interest due to the temporal distortion caused by use of phononic crystals. A complete ultrasound image requires beamforming to account for diffraction and focusing in the elevation plane in addition to the pulse-echo delays necessary to accurately reconstruct depth to a scattering event. The presence of strong frequency dispersion in phononic crystals causes the transient pulse to be temporally distorted as it passes through the lens. Distortion in the pulse shape decreases temporal fidelity and thus the axial resolution without additional signal processing. For practical, non-CW monostatic application, temporal distortion must be adequately addressed in addition to the contrast that is ideally a function of frequency-dependent backscattering^43^. Deconvolution to abate temporal pulse distortion with specialized pulse envelopes is left for future work. Here, the focus is the significant increase in lateral detection capabilities at depth in practice for both hard and soft material samples.

In conclusion, the application of a metamaterial to ultrasound imaging beyond the evanescent field and in simulated biosystems is reported. The lens is composed of a phononic crystal that combines elements only found in metamaterials to achieve collimation at narrow bandwidths of frequencies far above the homogenization limit of the phononic structure. The collimation-like effect results in a beam ~1.27*λ* in lateral width with negligible spatial dispersion from the end of the lens past the acoustic nearfield of the emitter. Both the acuity and lateral resolution of the ML were experimentally evaluated and compared to both a focused and planar transducer at the focal zone and end of the Fresnel zone in water ambient and gelatin tissue phantoms.

Compared with existing beamforming techniques, the azimuthal resolution and minimum separable distance is superior to the beamforming techniques of DAS, SA, and pixel-based beamforming. Though the ML does not exceed the FWHM of the beamforming technique of MV, the ML improvements in resolution is realized in less than ideal settings with no advanced signal processing. In addition, the measured results presented here are at the edge of the Fresnel zone for the utilized transducer. Focusing with transducers can ideally produce regions with similar beamwidths to the ML. However, achieving these minimum beamwidths comes at a cost of depth as focusing is a function of bringing the Fresnel–Fraunhofer transition zone closer to the emission source. The significant impact being that the ML maintains subwavelength azimuthal resolution at depth and along an extended depth of field that has not been achieved with existing beamforming techniques. Collimation from the ML results in is 8× better resolution than the TA at the Fresnel zone limit.

## Methods

### Metamaterial lens

The ML is a 2D phononic crystal shaped in a convex-triangular geometry operating in water ambient. The square lattice phononic crystal contains stainless steel rod scatterers with a radius, *r*, of 0.8 mm, and lattice constant, *a*, of 1.96 mm. The incident side of the lens is convex-plano, attached directly to a triangle shape with a base of 17*a* and height 9*a*. Details on the development of the design are given in other works^36^. However, the design is summarized as a combination of lens geometry, non-linear dispersion, and effective negative index of refraction for some angles of incidence to produce a spatially collimated beam. Collimation was only found for select frequency ranges using a collimation scoring algorithm^36^. The selection of the triangular design was based on it producing the narrowest beam waist with collimation over distance. The lack of beam spreading over distance grants the ML an advantage over conventional ultrasonic equipment for lateral resolving capabilities, particularly at depth.

### Bistatic measurements

We examined the lateral resolving capabilities of the lens in water ambient and tissue phantoms in both a bistatic and monostatic arrangement. A 1” Panametrics V301, single element, unfocused immersion transducer was used as the emitter in both arrangements. Bistatic measurements consisted of continuous wave sweeps from 510 to 590 kHz using a 2012 WaveStation function generator. For bistatic measurements, a Muëller-Platte 0.5 mm Needle Probe hydrophone served as the detector. For lateral scans, the hydrophone was aligned with the optical axis of the lens. Samples were then placed on a translating stage and swept the sample between the lens and hydrophone. Measurements were taken both with and without the lens for comparison.

### Monostatic measurements

Monostatic pulse-echo reflection spectroscopy measurements were performed with an Imaginant DPR300 Pulser-Receiver connected to the emitter. Data were acquired with the lens fixed, and the examined samples swept in front of the lens using a laterally translating stage. All measurements were performed in water ambient at room temperature. All data were collected on a Tektronix MDO3024b Spectrum Analyzer. Pulsing data consisting of 512 averaged signal samples with frequency information derived from a fast-Fourier transform (FFT) using a Hanning window. The sample was swept in front of the lens and the reflected signal recorded.

### Plastic rods (S1, S2)

3D printing was used to fabricate various ABS rods to evaluate ML acuity and lateral resolution. The rods were printed using a Polyprinter 229 and Hatchbox Acrylonitrile butadiene styrene (ABS) filament with a 0.2 mm diameter printing nozzle under 100% infilled setting. Sample 1 (S1, Fig. 2a) consisted of four rods spaced 6 mm apart with diameters ranging from 3 to 6 mm at 1 mm intervals. Sample 2 (S2, Fig 2b–f) was five pairs of 5 mm diameter rods with gaps of 5 mm, 4 mm, 1.90 mm (0.74*λ*), 1.30 mm (0.50*λ*), and 1.13 mm (0.44*λ*). Both S1 and S2 were in water ambient. For each measurement, the emitter and lens were fixed and the samples swept in front of the lens 60 mm from the emitter. The reflection was measured versus sample position and plotted. Figures 2a, b illustrate S1 and S2.

### Tissue-like phantoms (S3–S6)

Gelatin tissue phantoms were made following^33^. The gelatin was made using 1700 ml of water, in which 21 small bags (147 g) of KNOX were dissolved with a magnetic stirrer. Six boxes were added to the water while heating (before reach 50 °C) and then, the rest of the boxes were gradually added until it was perfectly mixed and achieved a viscous and homogeneous solution; the procedure took 90 min. After that, the gelatin solution was placed in a container and cooled for 24 h in a refrigerator. For measurements without objects inside, the gelatin was extracted from the container and cut in pieces of 55 mm high, 60 mm wide and 12 mm long. Speed of sound measurements for tissue phantoms and water were nearly identical at 1490 m/s in water, and 1494 m/s respectively. The dynamic bulk modulus and attenuation were found to be 2.775 GPa and 2.75 dB/cm for our samples.

Sample 3 (S3, Fig. 4a) consisted of hard objects in a tissue phantom. Three spherical plastic beads 5, 5, and 7 mm in diameter were placed into a tissue phantom of 55 mm high, 60 mm wide and 12 mm long during the synthesis process. The beads were spaced 5 mm between the two 5 mm beads, and 7 mm between the 5 and 7 mm beads in the pre-gelatinized solution. The objects were held by a 0.3 mm wires in order to keep those well attached in the gelatin and the solution was allowed to harden around the objects. Figure 4a is an image of S3.

Sample 4 (S4, Fig. 4c) was made by embedding a 7 mm × 6 mm × 16 mm rectangular prism of baby carrot (*Daucus carota)*, a 6.5 mm × 6 mm × 16 mm rectangular prism of celery (*Apium graveolens*), three stacked, tear-drop shaped, red apple seeds (*Pyrus malus L*.), and an 8 mm diameter Yaupon berry (*Ilex vomitoria*) in a tissue phantom (During the synthesis process the biomasses were set with a CTC distance of 13 mm between neighboring objects. An image of S4 is given in Fig. 4c.

Sample 5 (S5) consisted of four carrot mass slivers cut to widths of 1, 2, 3, and 4 mm prior to placement in the pre-gelatinized tissue phantom solution (Fig. 5a). The samples were placed at a depth of 25 mm in a 180 mm × 80 mm × 28 mm phantom tissue block ~30 mm apart. Sample 6 (S6) consisted of two carrot masses 6 mm × 4 mm × 4 mm and 4 mm × 4 mm × 4 mm placed 25 mm into the tissue phantom separated by 8 mm CTC (Fig. 5c).

To evaluate the spatial separation resolving capabilities of the ML in a monostatic arrangement, four pairs of carrots, ~7 mm in width were placed with in the same tissue phantom (Sample 7, S7, Fig. 6a). The masses were placed at 30 mm depth in the tissue phantom, and the gap between the paired biomasses set to 4 mm (1.44*λ*), 3 mm (1.08*λ*), 2 mm (0.72*λ*), and 1 mm (0.36*λ*).

Comparison of the ML to a focused transducer was performed using Sample 8 (S8, Fig. 7a). Carrot tissue masses were prepared using the same preparation as S5. However, masses are cut 1.5, 2, 3, 4, and 6 mm in width prior to placement 15-mm deep into the tissue phantom. Average separation between masses is 27 mm. It should be noted that the 1.5 mm masses was displaced by 10 mm during synthesis so that its depth is only 5 mm into the tissue phantom. It is still presented for reference as both the ML and focused transducer were applied equally.

## Data Availability

Data available upon request from either corresponding author.

## Code availability

Code available upon request from either corresponding author.
